# Murine Colitis Modeling using Dextran Sulfate Sodium (DSS)

**DOI:** 10.3791/1652

**Published:** 2010-01-19

**Authors:** Caitlyn G. Whittem, Amanda D. Williams, Christopher S. Williams

**Affiliations:** Department of Cancer Biology, Vanderbilt University; Departments of Medicine and Cancer Biology, Vanderbilt University

## Abstract

Colitis can occur from viral or bacterial infections, ischemic insult, or autoimmune disorders; most notably Ulcerative Colitis and the colonic variant of Crohn’s Disease - Crohn’s Colitis.  Acute colitis may present with abdominal pain and distention, malabsorption, diarrhea, hematochezia and mucus in the stool.  We are beginning to understand the complex interactions between the environment, genetics, and epithelial barrier dysfunction in Inflammatory Bowel Disease and animal models of colitis have been essential in advancing our understanding of this disease.  One popular model involves supplementing the drinking water of mice with low-molecular weight Dextran Sodium Sulfate (DSS), resulting in epithelial damage and a robust inflammatory response in the colon lasting several days ^1^.Variations of this approach can be used to model acute injury, acute injury followed by repair, and repeated cycles of DSS interspersed with recovery modeling chronic inflammatory diseases ^2^. After a single four-day treatment of 3% DSS in drinking water, mice show signs of acute colitis including weight loss, bloody stools, and diarrhea.  Mice are euthanized at the conclusion of the treatment course and at necropsy dissected colons are processed and can be 'Swiss rolled" ^3^ to allow microscopic analysis of the entire colon or infused with formalin as "sausages" to allow macroscopic analysis. Tissue is then embedded in paraffin, sectioned, and stained for histologic review.

**Figure:**
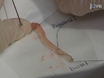


## Protocol

### Part 1:  Injury-Repair Colitis Model

Baseline weight should be obtained for each mouse prior to beginning DSS treatment.
Make a 3% (w/v) Dextran Sulfate Sodium Salt solution in water and filter with a 0.45 μm cellulose acetate filter.
Replace drinking water in the mouse cage with the 3% DSS solution for four days.  The mice should not have access to any other source of water (i.e. exclusion tips placed on automated watering systems).
On day four, replace the DSS solution with water for an additional three days, allowing some colonic epithelial recovery.  The mice should be weighed on day four in order to quantify systemic consequences of colitis. Weight loss is common with severe injury.
On day 7, weigh and sacrifice the mice.  Mice can be euthanized by inhalational overdose of isoflurane, or other institutionally, IACUC approved methods.

### Part 2:  Necropsy and Harvesting of Colon

Expose the ventral side of the mouse and secure legs to ensure unobstructed access to the abdomen.  Wet the abdomen completely with 70% ethanol.
Grasp the midline of the abdomen with tissue forceps and extend upwards thus tenting the skin. Use fine-tipped scissors to incise the abdomen thus exposing the abdominal contents. Extend the incision to the tip of the xyphoid process at the midline and then extend along the inferior aspect of the costal margins bilaterally.
Identify the small intestine, cecum, and colon. Carefully dissect/tease the colon from the surrounding mesentery. Transect the colon deep in the pelvis to free the distal colon and rectum. Transect the colon at the colonocecal margin to free the proximal colon. Care must be taken during this process not to damage the colon as cleaning the colon will be problematic if perforation occurs.
Using a 20G feeding needle and 10 ml syringe, intubate and flush the colon with ice-cold PBS until the eluate is completely clear of stool.
At this point, if macroscopic analysis with a dissecting microscope is desired, the colons can be fixed as “sausages”. If histologic analysis only is desired than proceed to Part 4 “Making Swiss Rolls for the Histological Analysis of Acute Colitis”.

### Part 3:  Processing as “Sausages” for Macroscopic Analysis of the Entire Colon

It is important to maintain the correct orientation of the colon, therefore, keep the distal end of the colon closest to the operator. Cut two pieces of non-absorbable suture (SP116 1.5 metric braided silk), one approximately 1 inch in length, the other 2 inches.
Use the 2 inch piece of suture to tie off the distal end as close to the margin as possible while still maintaining a good seal.
Place a 20G feeding needle containing 5 ml of 10% buffered formalin phosphate into the proximal end of the colon. Loosely tie the remaining piece of suture immediately proximal to the bulb of the feeding needle. Grasp the colon firmly at the bulb of the feeding needle and infuse formalin until the colon is expanded. Tighten the knot in the suture as you withdraw the feeding needle, thus leaving the infused, expanded colon as a “sausage”.
Fill a 15 ml conical tube with 10% buffered formalin phosphate and place the colon in the tube.  Fix for 24 hours.
Pour off formalin and replace with 70% EtOH for an additional 24hrs. (Colons can be stored in 70% EtOH indefinitely at room temperature.)
Remove the colon from the conical tube and cut the strings on each end with a scalpel, being careful not to damage the colon.  Remember that the distal end has the longest piece of string.  Cut longitudinally from the distal to proximal end of the colon so that it forms a long sheet.  At this point, the colon can be viewed under a dissecting microscope.

### Part 4:  Making Swiss Rolls for the Histological Analysis of Acute Colitis

It is important to maintain the correct orientation of the colon, therefore, keep the distal end of the colon closest to the operator.
Measure the colon length. This metric is another indicator of the severity of injury. Colitis increases edema and shortens the overall colon length.
Using fine-tipped scissors, incise longitudinally from distal to proximal end of the colon. Use fine tipped forceps to grasp either edge of the incision and open laterally working your way distally->proximally, thus displaying the colon as a flat sheet.
Rolling the colon requires a pair of forceps and a two handed technique. Grasp either edge of the distal margin with forceps. Proceed to sequentially roll the colon by rotating and releasing the forceps proceeding to the proximal margin. Thus generating a spiral with a third dimension or “Swiss Roll”.  To maintain the rolled form, grasp the roll firmly with forceps and transect it with a 27G1/2 needle. Secure the needle by bending the needle as it exits the roll.
Place the roll in an appropriately labeled tissue cassette and place in a jar of 10% buffered formalin phosphate at room temperature for 24 hrs to ensure tissue fixation.
Pour off formalin and replace with 70% EtOH for an additional 24hrs. (Colons can be stored in 70% EtOH indefinitely at room temperature.)
Once the sample has been fixed, it can be paraffin-embedded, sectioned and mounted on slides for histologic analysis.

### Part 5: Representative Results

The DSS model for acute colitis allows the researcher to obtain, fix, and analyze a colon that models acute colitis.  When the Swiss roll is cut and mounted, it should form a representative slice of the entire colon if rolled properly (Figure 1).  The mounted roll can be stained with H&E in order to determine the extent of damage to the colon, from the distal (inside) end to the proximal (outside) end (Figure 2).  Immunohistochemistry can also be performed on the Swiss roll to identify and quantify inflammatory infiltrates.  If the sausage method has been performed correctly, the fixed colon will be dilated and the entire mucosal surface can be easily manipulated and viewed under the dissecting microscope (Figure 3).


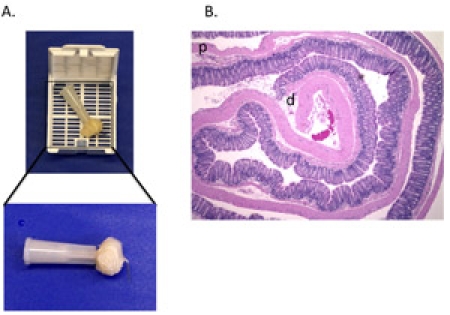
 Click here to see a larger Figure. **Figure 1.**  The properly-executed "Swiss roll".  (A) The colon is rolled from the distal to proximal end, transected with a needle and secured by bending the end of the needle. It is then placed in  a tissue cassette for fixation.  (B) H&E stained 5 μm section of a Swiss roll made from the colon of a mouse treated with DSS (d= distal colon p= proximal colon).


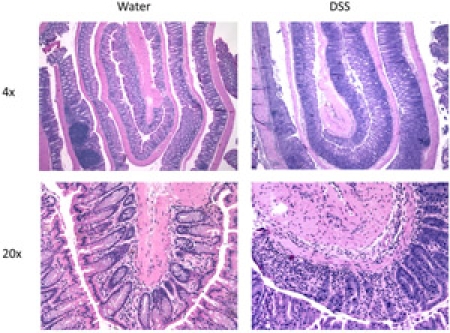
 Click here to see a larger Figure **Figure 2.**  DSS treated colons show signs of acute colitis.  Inflammation and crypt damage are apparent in the DSS-treated colon compared to a water treated control.


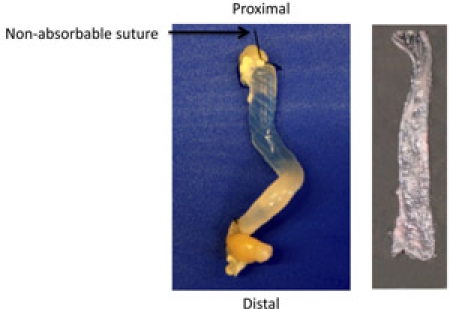
 Click here to see a larger Figure **Figure 3. ** An example of a "sausage".  The sausage infused with formalin and completely expanded.  A slight angle will be present secondary to the natural curvature of the colon.  The opened sausage should lie flat.

## Discussion

This protocol can be modified to model acute injury, injury-repair, or chronic colonic injury processes.

### Acute Injury Modification:

DSS treatment ad lib for 4-6 days followed by immediate sacrifice

### Injury-Repair Modification:

Injury with 4-6 days of DSS treatment followed by recovery period of 3-4 days and sacrifice (as described in above protocol).

### Chronic Colitis Modification:

Mice are placed on three five-day cycles of 3% DSS with sixteen days of recovery between each cycle.  Mice are sacrificed after the final 16-day rest period.

### There are several issues that researchers need to be aware of with this model:

Variability in site of injury: In our hands, we see a predominantly distal injury pattern, with relative sparing of the proximal colon and cecum.  Others have reported more proximal predominance to the injury pattern.
Environmental variability. There is significant environmental variability. This is likely, in part, due to differences in intestinal microflora, diet and other environmental considerations.
This model is ineffective if high molecular weight DSS is used (i.e. 500,000 Da).
There is considerable strain variability with this model ^4^.

Biochemical analysis of the colon can be performed by taking samples from the proximal or distal margins. Depending on the severity of injury this may impact your ability to generate a histologic injury score. In vivo BrdU labeling to measure proliferation can be achieved by intraperitoneal injection of 16.7 μg/kg BrdU 2 hrs prior to sacrifice followed by α-BrdU IHC processing.

As an alternative to the “sausage”, the colon can be fixed completely flat.  Line the bottom of a Tupperware container with Whatman paper and soak the paper with 10% buffered formalin phosphate.  Dissect the colon as described in “Part 2: Necropsy and Harvesting of Colon”.  Again, keep the distal end of the colon closest to the operator. Using fine-tipped scissors, incise longitudinally from distal to proximal end of the colon. With fine tipped forceps grasp either edge of the incision and open laterally working, thus displaying the colon as a flat sheet.  Transfer this flat sheet to the pre-soaked Whatman paper.  Cover the flattened colon with another piece of Whatman and wet with 10% buffered formalin phosphate.  The Whatman should be completely covered in formalin but not so much that it lifts off the colon.  Seal the Tupperware and fix for 24 hours.  Remove the colon from the Tupperware and place it in 10 ml 70% ethanol in a 15 ml conical tube. (Colons can be stored in 70% EtOH indefinitely at room temperature.)

There are several methods for quantifying colonic injury ^5,6,7^. One method, for example, uses a multi-parameter scale including: inflammation, extent involvement, regeneration, crypt damage, and percent involvement ^8^.
